# Supplementary material for: Transcriptional repression of beige fat innervation via a YAP/TAZ-S100B axis
Source: Nat Commun. 2023 Nov 4;14:7102. doi: 10.1038/s41467-023-43021-8 (PMC10625615; doi:10.1038/s41467-023-43021-8)
Supplement: Supplementary file 1 — Supplementary Information [file 41467_2023_43021_MOESM1_ESM.pdf]

Transcriptional repression of beige fat innervation via a YAP/TAZ-S100B axis

Xun Huang[1,2,3], Xinmeng Li[1], Hongyu Shen[1,2,3], Yiheng Zhao[1,2,3], Zhao Zhou[1], Yushuang Wang[1], Jingfei Yao[1], Kaili Xue[1], Dongmei Wu[1,2*], Yifu Qiu[1,2*]

[1]Institute of Molecular Medicine, Beijing Key Laboratory of Cardiometabolic Molecular Medicine, College of Future Technology, Peking University, Beijing 100871, China.

[2]Peking-Tsinghua Center for Life Sciences, Peking University, Beijing 100871, China.

[3]Academy for Advanced Interdisciplinary Studies, Peking University, Beijing 100871, China.

*Correspondence: dongmei.wu@pku.edu.cn; yifu.qiu@pku.edu.cn

Supplementary Figure 1

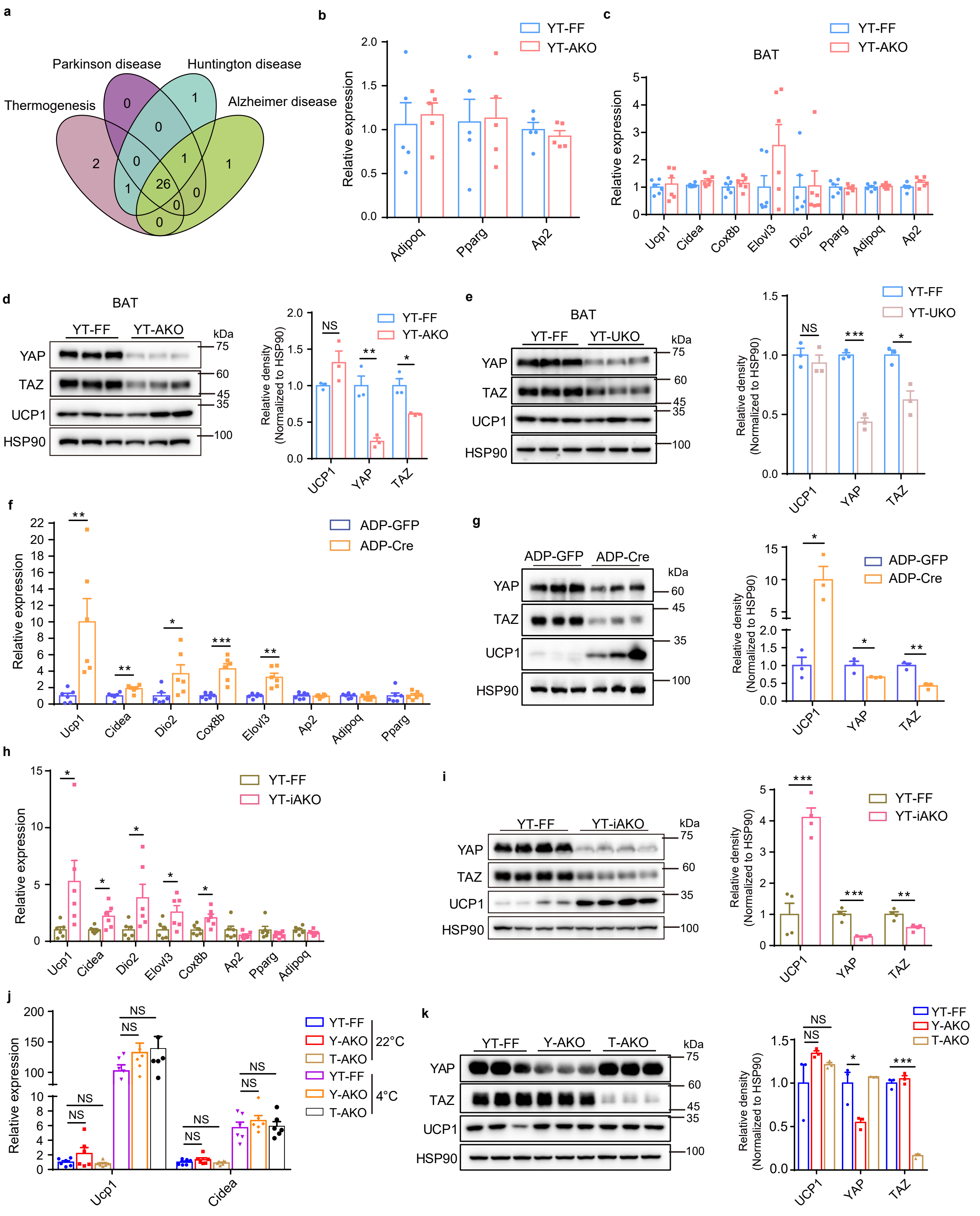

Supplementary Figure 1. **Double knockout of *Yap* and *Taz* promotes scWAT thermogenesis**. **a**, A Venn diagram showing the common genes shared by GO terms, including thermogenesis, Parkinson disease, Huntington disease and Alzheimer disease. **b**, Relative mRNA levels of adipogenic genes in scWAT. **c**, Relative mRNA levels of thermogenic genes in BAT. **d**, Immunoblot of indicated proteins in BAT as in **c**. **e**, Immunoblot of indicated proteins in BAT of YT-UKO mice. **f**, Relative mRNA levels of thermogenic and adipogenic genes of YT-FF scWAT injected with AAV-ADP-Cre or AAV- ADP-GFP for 4 weeks. **g**, Immunoblot of indicated proteins in scWAT as in **f**. **h**, Relative mRNA levels of thermogenic and adipogenic genes in scWAT from 12-week-old YT-iAKO or YT-FF male mice intraperitoneally injected with tamoxifen for 4 weeks. **i**, Immunoblot of indicated proteins in scWAT as in **h**. **j**, Relative mRNA levels of *Ucp1* and *Cidea* in scWAT from Y-AKO, T-AKO and YT-FF mice. **k**, Immunoblot of indicated proteins in scWAT as in **j**. N=3 (**d**, **e, g** and **k**), 4 (**i**), 5 (**b**) or 6 (**c**, **f**, **h** and **j**) mice per group, and data are mean ± s.e.m. Two-tailed unpaired Student's *t*-test (**b-i**); one-way ANOVA with Bonferroni's multiple comparisons test (**j** and **k**). *$P < 0.05$, **$P < 0.01$, ***$P < 0.001$; NS, not significant. Specific p-values and source data are provided as a Source Data file.

Supplementary Figure 2

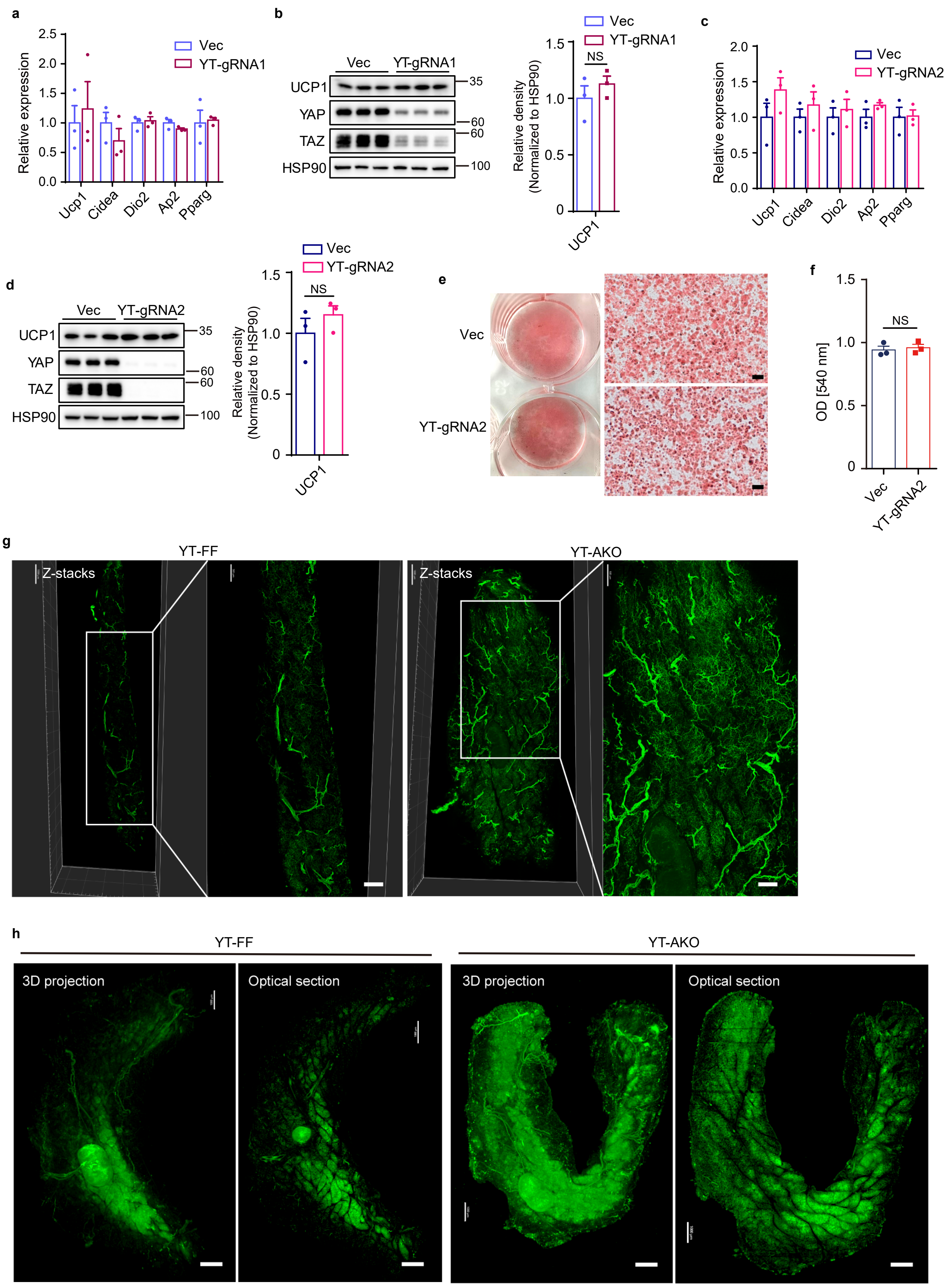

Supplementary Figure 2. ***Yap/Taz*** **deficiency promotes sympathetic innervation in scWAT and has no effect on adipocyte identity**. **a**, **c**, Relative mRNA levels of indicated genes in SVF-derived beige adipocytes from Cas9$^{tg/tg}$ scWAT injected with AAV-Vec and AAV-*Yap*/*Taz*-gRNA1 (**a**) or AAV-*Yap*/*Taz*-gRNA2 (**c**). **b**, **d**, Immunoblot of indicated proteins in SVF-derived beige adipocytes as in (**a**) and (**c**). **e**, **f**, Oil Red O staining (**e**) and quantification (**f**) of SVF-derived beige adipocytes as in (**c**). Scale bars: 100 μm. **g**, Z-stack (200 μ m) images of whole-mount TH immunostaining as in Fig 3 (**a**). Scale bars: 0.5 mm. **h**, Whole-mount UCP1 immunostaining and optical sections of cleared scWAT from YT-AKO and YT-FF mice. Scale bars: 1 mm. N=3 (**a-d** and **f**) biologically independent cell cultures per group, and data are mean ± s.e.m. Two-tailed unpaired Student's *t*-test (**a-d** and **f**). *P < 0.05, **P < 0.01, ***P < 0.001; NS, not significant. Specific p-values and source data are provided as a Source Data file.

Supplementary Figure 3

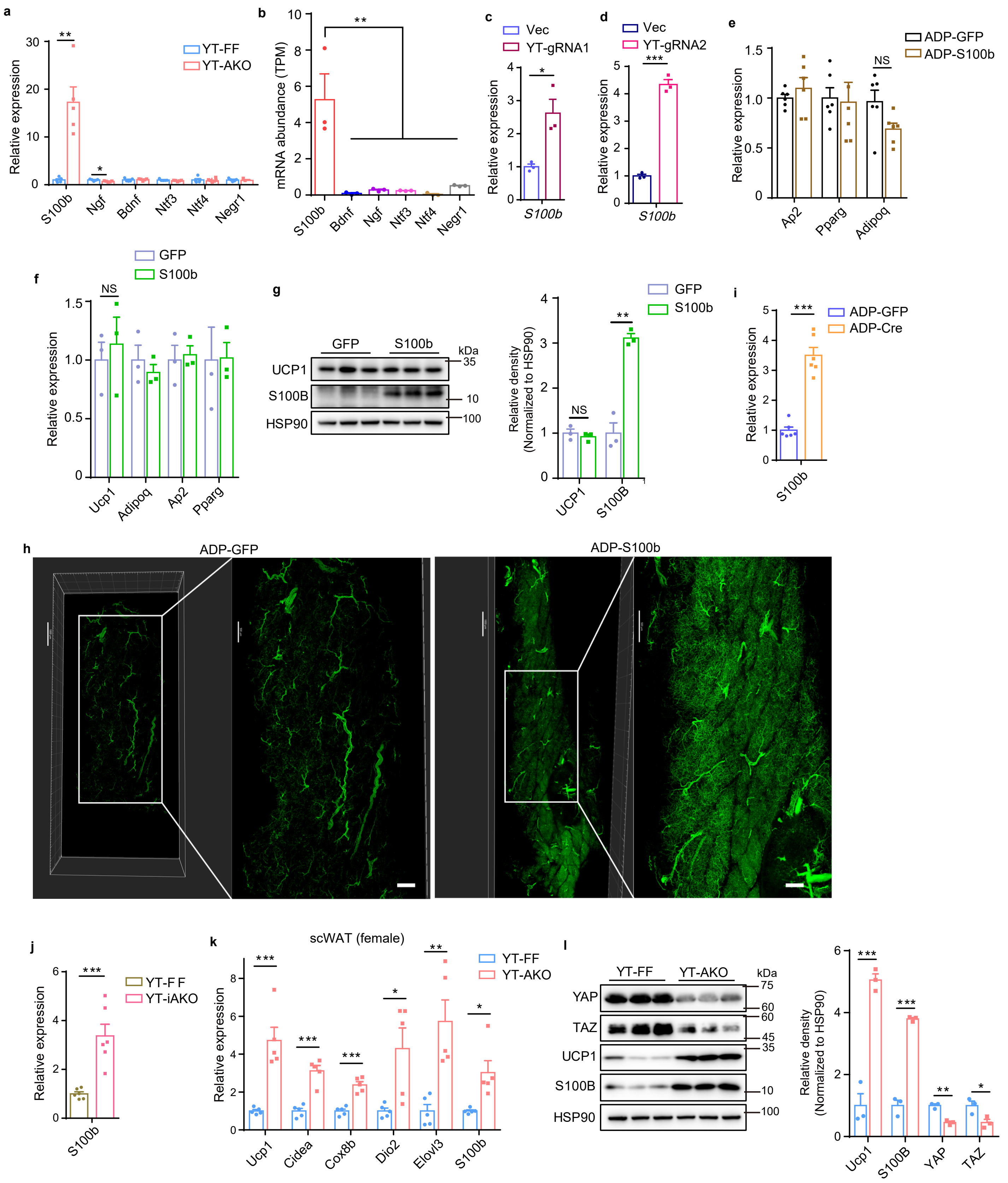

Supplementary Figure 3. ***Yap*/*Taz* deficiency in adipocytes increases S100b expression and innervation**.

a, Relative mRNA levels of indicated neurotrophic genes in scWAT. **b**, mRNA abundance (TPM) of indicated neurotrophic genes in scWAT from wild-type mice, measured by RNA-seq. **c**, **d**, Relative mRNA levels of S100b in SVF-derived beige adipocytes as in Supplementary Fig. 2 (**a**) and (**c**). **e**, Relative mRNA levels of adipocyte characteristic genes in scWAT from wild-type mice ectopically expressed with S100b and GFP. **f**, **g**, Relative mRNA (**f**) and protein (**g**) levels of indicated genes in SVF-derived beige adipocytes enforcedly expressed with S100b and GFP. **h**, Z-stack (200μm) images of whole-mount TH immunostaining as in Fig 3 (**i**). Scale bars: 0.5 mm. **i**, Relative mRNA level of *S100b* in YT-FF scWAT injected with AAV-ADP-Cre or AAV- ADP-GFP for 4 weeks. **j**, Relative mRNA level of *S100b* in scWAT from 12-week-old YT-iAKO or YT-FF male mice injected intraperitoneally with tamoxifen for 4 weeks. **k**, **l**, Relative mRNA (**k**) and protein (**l**) levels of indicated genes in scWAT from 8-week-old female YT-AKO and control mice. N=3 (**c**, **d**, **f** and **g**) biologically independent cell cultures per group and n=3 (**b** and **l**), 5 (**a** and **k**) or 6 (**e**, **i** and **j**) mice per group, and data are mean ± s.e.m. Two-tailed unpaired Student's *t*-test (**a**, **c**-**g**, and **i**-**l**); one-way ANOVA with Bonferroni's multiple comparisons test (**b**). *$P < 0.05$, **$P < 0.01$, ***$P < 0.001$; NS, not significant. Specific p-values and source data are provided as a Source Data file.

Supplementary Figure 4

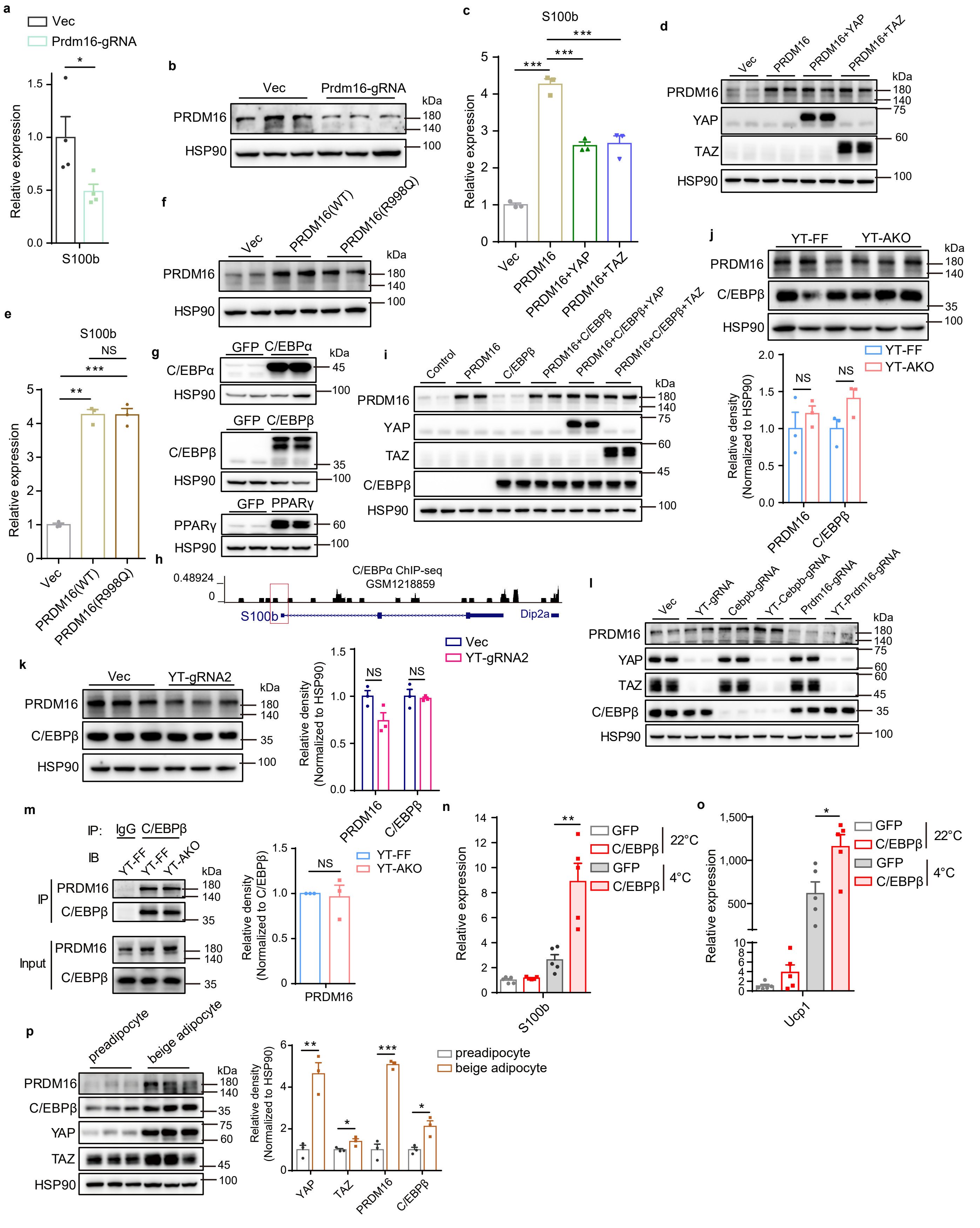

Supplementary Figure 4. PRDM16 and C/EBPβ promote S100b expression. a, Relative mRNA level of *S100b* in *Rosa26-LSL-Cas9;Adipoq^{Cre}* scWAT injected with AAV-*Prdm16*-gRNA for 4 weeks. **b**, Immunoblot of PRDM16 in scWAT in (**a**). **c**, Relative mRNA level of *S100b* in SVF-derived beige adipocytes expressed with indicated proteins. **d**, Immunoblot of indicated proteins in SVF-derived beige adipocytes in (**c**). **e**, Relative mRNA level of *S100b* in SVF-derived beige adipocytes expressed with PRDM16 or DNA-binding-deficient PRDM16 (R998Q). **f**, Immunoblot of PRDM16 in SVF-derived beige adipocytes in (**e**). **g**, Immunoblot of indicated proteins in SVF-derived beige adipocytes expressed with C/EBPα, C/EBPβ and PPARγ respectively. **h**, Alignment of C/EBPα ChIP-seq peaks at *S100b* gene locus in brown adipocytes. **i**, Immunoblot of indicated proteins in SVF-derived beige adipocytes expressed with indicated proteins. **j**, Immunoblot of indicated proteins in scWAT from 8-week-old YT-FF and YT-AKO mice. **k**, Immunoblot of indicated proteins in SVF-derived beige adipocytes from Cas9$^{tg/tg}$ scWAT injected with AAV-Vec and AAV-*Yap*/*Taz*-gRNA2. **l**, Immunoblot of indicated proteins in SVF-derived beige adipocytes from Cas9tg/tg scWAT injected with AAV-Vec, AAV-Yap/Taz-gRNA, AAV-Cebpb-gRNA, AAV-Yap/Taz-Cebpb-gRNA, AAV-Prdm16-gRNA, AAV-Yap/Taz-Prdm16-gRNA respectively. **m**, Endogenous co-IP of PRDM16 and C/EBPβ and quantification of immunoprecipitated PRDM16 in BAT from YT-AKO and YT-FF adult mice. **n**, **o**, Relative mRNA level of *S100b* (**n**) and *Ucp1* (**o**) in scWAT injected with AAV-ADP-C/EBPβ or AAV-ADP-GFP for 4 weeks under 22°C and 4°C for 2 days. **p**, Immunoblot and quantification of indicated proteins in scWAT-derived SVF before and after

differentiation. N=3 (**j** and **m**), 4 (**a**) or 5 (**n** and **o**) mice per group and n=3 (**b**, **c**, **e**, **k** and **p**) biologically independent cell cultures per group, and data are mean ± s.e.m. Two-tailed unpaired Student's *t*-test (**a**, **c**-Vec versus PRDM16 group, **j**, **k** and **m**-**p**); one-way ANOVA with Tukey's multiple comparisons test (**c** and **e**). $^{*}P < 0.05$, $^{**}P < 0.01$, $^{***}P < 0.001$; NS, not significant. Specific p-values and source data are provided as a Source Data file.

Supplementary Figure 5

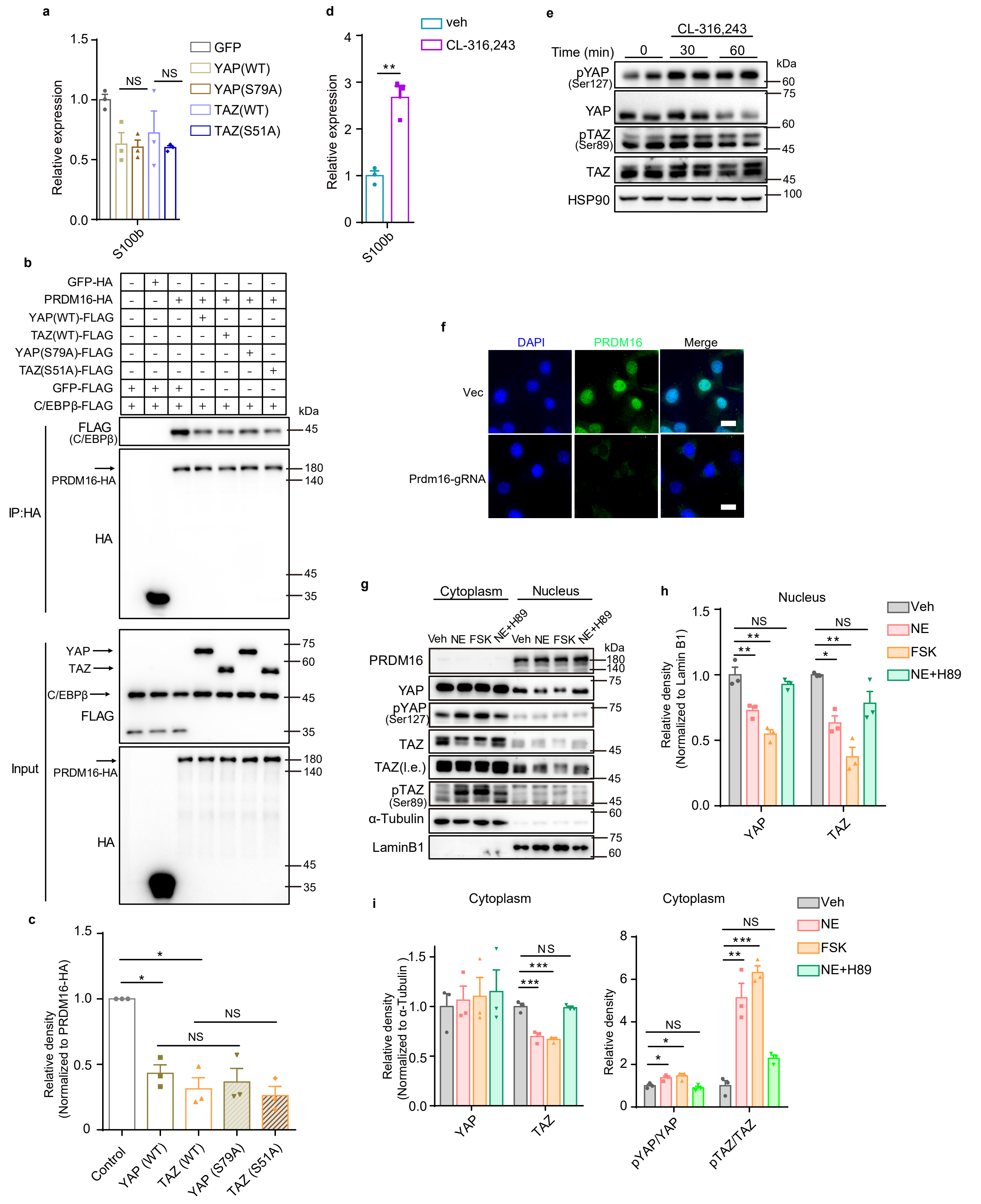

Supplementary Figure 5. NE-cAMP-PKA signaling stimulates YAP/TAZ phosphorylation and excludes them from the nucleus. **a**, Relative mRNA level of *S100b* after expression with YAP, TEAD-binding-deficient YAP (S79A), TAZ, TEAD-binding-deficient TAZ (S51A) and GFP respectively. **b**, **c**, Co-IP of C/EBPβ and PRDM16 (**b**) and quantification of immunoprecipitated C/EBPβ (**c**) in HEK-293T cells expressed with indicated proteins (n=3 biologically independent experiments). **d**, Relative mRNA level of *S100b* after treatment with CL-316,243 for 12 hours. **e**, Phosphorylation of YAP and TAZ after treatment with CL-316,243. **f**, Immunofluorescence of PRDM16 in scWAT-derived beige adipocytes infected sequentially with Cas9 lentivirus and vec or Prdm16-gRNA AAV. Scale bars: 20 μm. **g**-**i**, Immunoblot (**g**) and quantification (**h**, **i**) of indicated proteins in cytoplasm and nucleus fraction after treatment with indicated reagents for 30 minutes. Experiments in (**a**, **d**, **e**, **g**) were performed in SVF-derived beige adipocytes. N=3 (**a**, **d**, **h** and **i**) biologically independent cell cultures per group, and data are mean ± s.e.m. Two-tailed unpaired Student's *t*-test [**d**, Veh versus FSK groups in (**h**) and (**i**)]; Two-tailed paired Student's *t*-test (**c**); one-way ANOVA with Tukey's multiple comparisons test (**a, h** and **i**). $^{*}P < 0.05$, $^{**}P < 0.01$, $^{***}P < 0.001$; NS, not significant. Specific p-values and source data are provided as a Source Data file.

Supplementary Figure 6

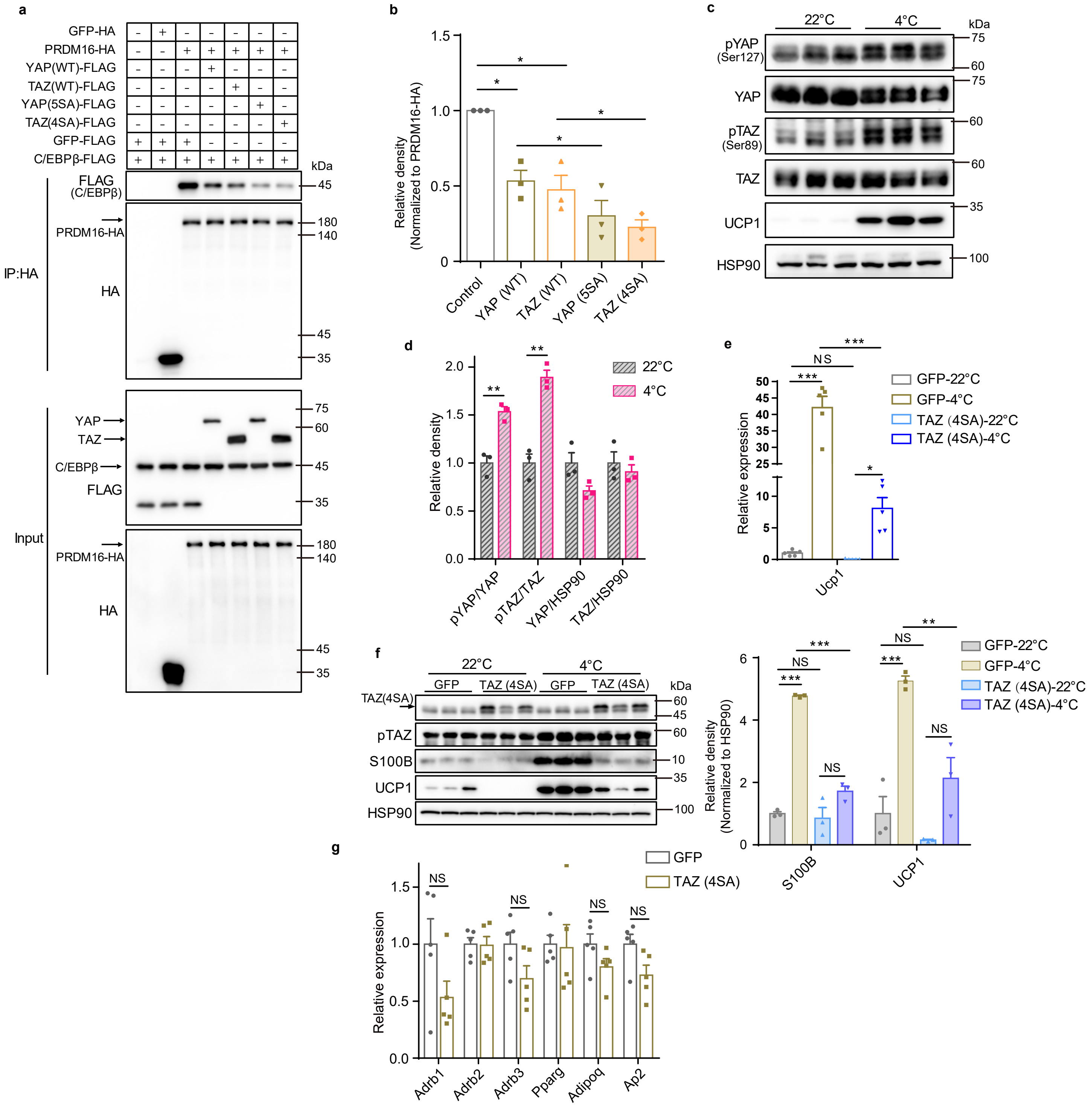

Supplementary Figure 6. Non-phosphorylated YAP/TAZ repress cold-induced S100b expression. **a**, **b**, Co-IP of C/EBPβ and PRDM16 (**a**) and quantification of immunoprecipitated C/EBPβ (**b**) in HEK-293T cells expressed with indicated proteins (n=3 biologically independent experiments). **c**, **d**, Immunoblot (**c**) and quantification (**d**) of indicated proteins in scWAT under 4°C for 12 hours. **e**, Relative mRNA level of *Ucp1* in scWAT injected with AAV-ADP-TAZ(4SA) or AAV-ADP-GFP for 3 weeks then under 22°C or 4°C for 2 days. **f**, Immunoblot of indicated proteins in scWAT as in (**e**). **g**, Relative mRNA level of indicated genes in scWAT as in (**e**). N=3 (**d** and **f**) or 5 (**e** and **g**) mice per group, and data are mean ± s.e.m. Two-tailed unpaired Student's *t*-test (**d** and **g**); Two-tailed paired Student's *t*-test (**b**); two-way ANOVA with Tukey's multiple comparisons test (**e** and **f**). *$P < 0.05$, **$P < 0.01$, ***$P < 0.001$; NS, not significant. Specific p-values and source data are provided as a Source Data file.

Supplementary Figure 7

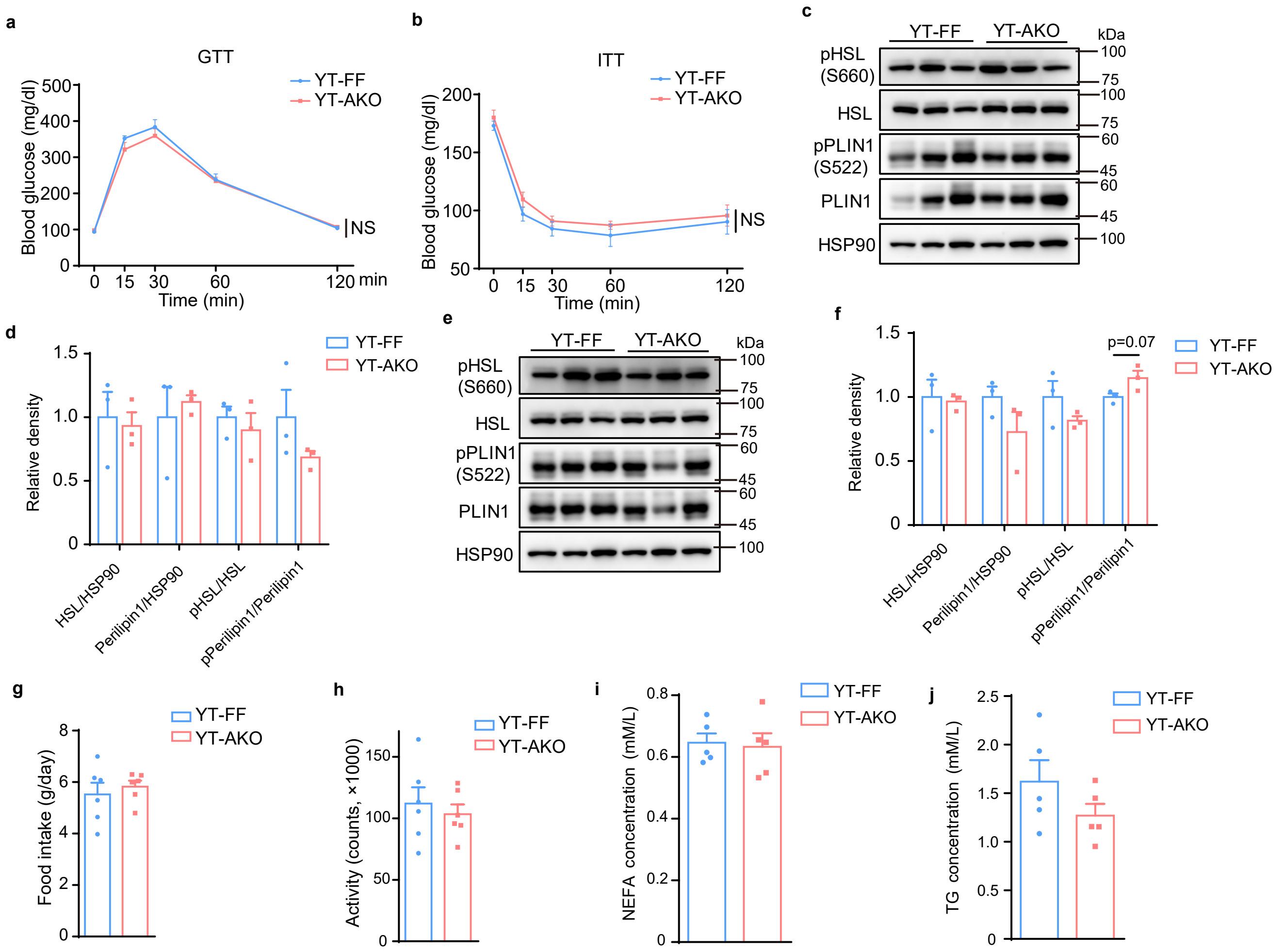

Supplementary Figure 7. Adipocyte-specific loss of *Yap/Taz* did not affect lipolytic ability in WAT. **a**, **b**, Glucose-tolerance test (GTT, **a**) and insulin-tolerance test (ITT, **b**) of 8-week-old YT-AKO and YT-FF mice. **c**, **d**, Immunoblot (**c**) and quantification (**d**) of indicated proteins in scWAT. **e**, **f**, Immunoblot (**e**) and quantification (**f**) of indicated proteins in eWAT. **g**, **h**, Food intake (**g**) and activity (**h**). **i**, **j**, NEFA and TG concentration in the sera. Experiments in (**c**-**j**) were performed in 12-month-old YT-AKO and YT-FF male mice. N=3 (**c**-**f**), 5 (**i** and **j**), 6 (**g** and **h**), 8 (**a** and **b**) mice per group, and data are mean ± s.e.m. Two-tailed unpaired Student's *t*-test (**d**, **f** and **g**-**j**); two-way ANOVA (**a** and **b**). *$P < 0.05$, **$P < 0.01$, ***$P < 0.001$; NS, not significant. Specific p-values and source data are provided as a Source Data file.

Supplementary Figure 8

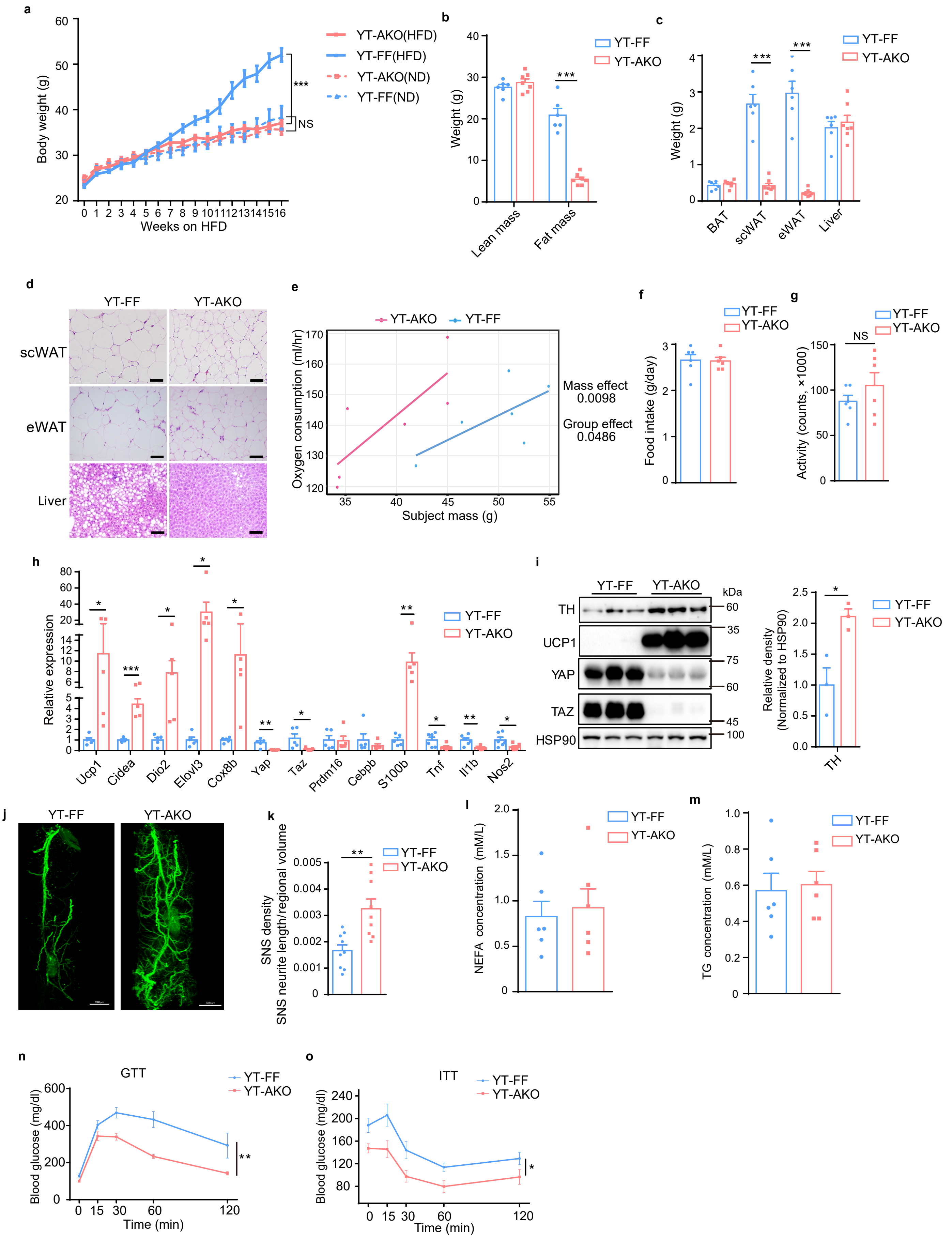

Supplementary Figure 8. Loss of *Yap/Taz* in adipocytes protects mice from diet-induced obesity and insulin resistance. **a**, Body weight of YT-AKO and YT-FF mice on HFD or ND. **b**, **c**, Fat mass and lean mass (**b**) and tissue weight (**c**) of YT-AKO and YT-FF mice on HFD for 16 weeks. **d**, H&E sections of scWAT, eWAT and liver from mice in **b**. Scale bars: 50 μm. **e**, Oxygen consumption of YT-AKO and YT-FF mice on HFD for 15 weeks, analyzed by CaIR-ANCOVA. **f**, **g**, Food intake (**f**) and activity (**g**) of YT-AKO and YT-FF mice on HFD for 15 weeks. **h**, Relative mRNA level of indicated genes in scWAT from mice as in **b**. **i**, Immunoblot of indicated proteins in scWAT as in **b**. **j, k,** Whole-mount TH immunostaining (**j**) and quantification (**k**) of cleared scWAT as in **b**. Scale bars: 2 mm. **l**, **m**, NEFA (**l**) and TG (**m**) concentration in the sera of YT-FF and YT-AKO mice fed HFD for 15 weeks. **n**, **o**, Glucose-tolerance test (GTT, **n**) and Insulin-tolerance test (ITT, **o**) of YT-AKO and YT-FF mice on HFD. N=3 (**i**), 5 (**h**), 6 (**b**, **c**: YT-FF and **e-g** and **l-o**), 7 (**a**: YT-AKO(ND) and **b, c**: YT-AKO), 8 (**a**: YT-FF(ND)) or 12 (**a**: YT-FF(HFD) and YT-AKO(HFD) mice per group, and data are mean ± s.e.m. Two-tailed unpaired Student's *t*-test (**b**, **c**, **f-m**); two-way ANOVA (**a**, **n** and **o**). $^{*}P < 0.05$, $^{**}P < 0.01$, $^{***}P < 0.001$; NS, not significant. Specific p-values and source data are provided as a Source Data file.

Supplementary Figure 9

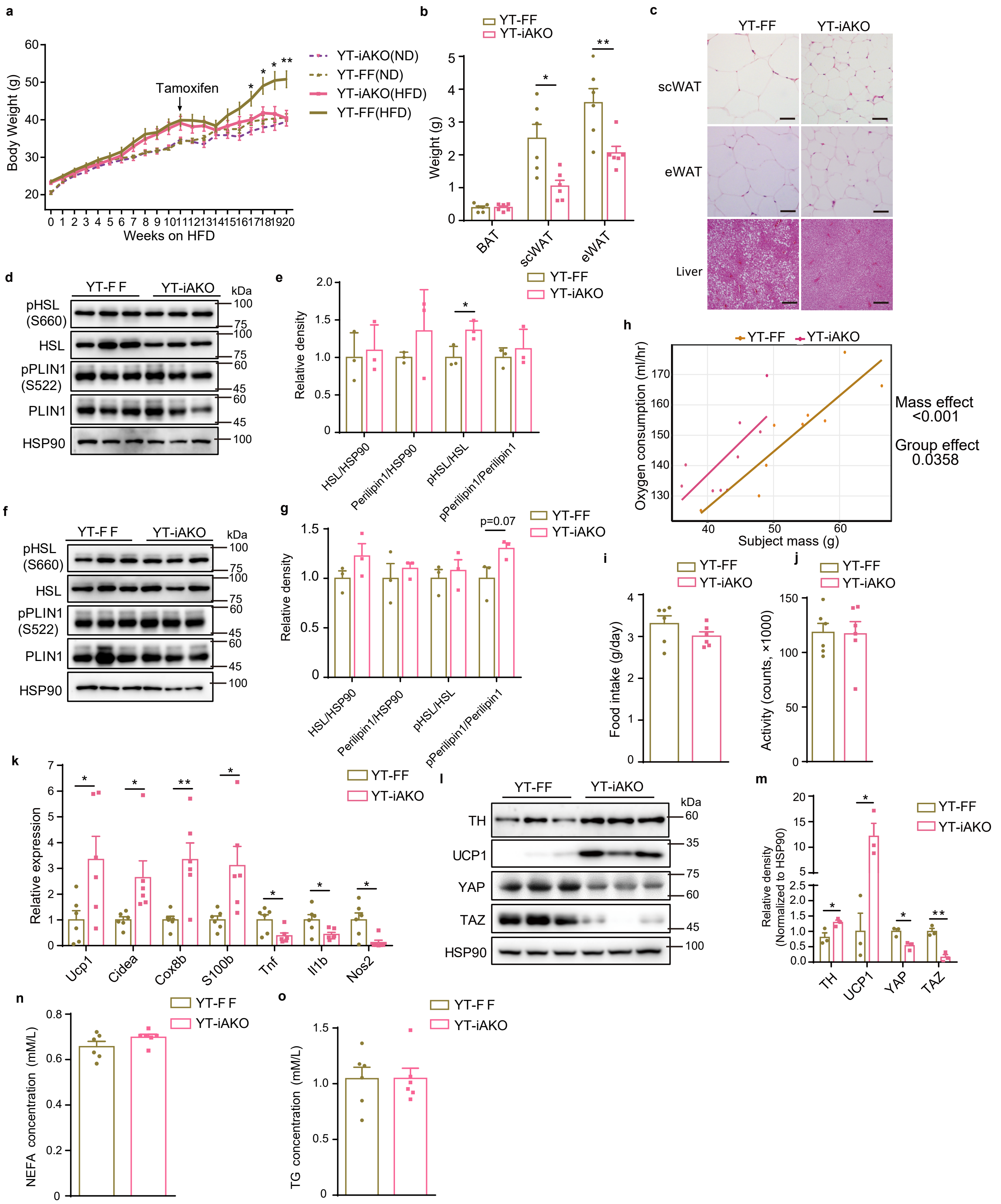

Supplementary Figure 9. Reversal of obesity by inducible knockout of adipocyte *Yap/Taz* in diet-induced obese mice. **a**, Body weight of YT-iAKO and YT-FF mice on HFD or ND. **b**, Tissue weight of YT-iAKO and YT-FF mice on HFD for 20 weeks. **c**, H&E sections of scWAT, eWAT and liver from mice in **b**. Scale bars: 50 μm. **d**, **e**, Immunoblot (**d**) and quantification (**e**) of indicated proteins in scWAT from mice as in **b**. **f**, **g**, Immunoblot (**f**) and quantification (**g**) of indicated proteins in eWAT from mice as in **b**. **h**, Oxygen consumption of mice in **b**, analyzed by CaIR-ANCOVA. **i**, **j**, Food intake (**i**) and activity (**j**) of YT-iAKO and YT-FF mice on HFD for 19 weeks. **k**, Relative mRNA levels of indicated genes in scWAT from mice as in **b**. **l**, **m**, Immunoblot (**l**) and quantification (**m**) of indicated proteins in scWAT from mice as in **b**. **n**, **o**, NEFA (**n**) and TG (**o**) concentration in the sera of mice as in **b**. N=3 (**d**-**g**, **l** and **m**), 6 (**a**-ND groups, **b**, **i**-**k**, **n** and **o**) or 9 (**a**-HFD group and **h**) mice per group, and data are mean ± s.e.m. Two-tailed unpaired Student's *t*-test (**b**, **e**, **g**, **i**-**k** and **m**-**o**); two-way ANOVA with Tukey's multiple comparisons test (**a**). $^{*}P < 0.05$, $^{**}P < 0.01$, $^{***}P < 0.001$. Specific p-values and source data are provided as a Source Data file.

Supplementary Figure 10

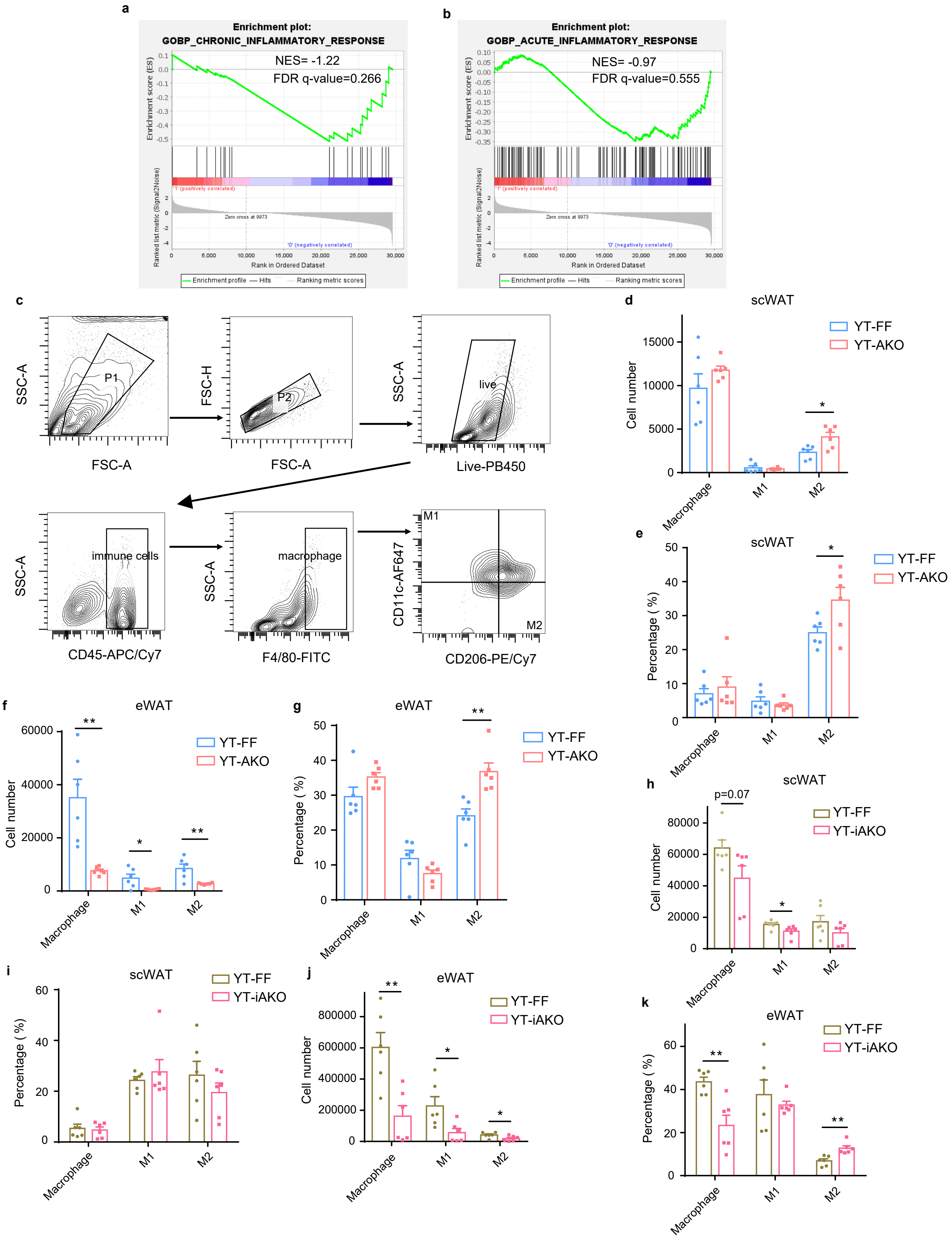

Supplementary Figure 10. **YAP/TAZ loss reduces inflammatory response in scWAT and eWAT in HFD-reduced or aging-associated obesity condition**. **a**, **b**, GSEA of chronic inflammatory response (**a**) and acute inflammatory response (**b**) based on RNA-seq data of scWAT from adult YT-FF and YT-AKO mice. **c**, Gating strategy of flow cytometry for macrophage phenotyping. **d**, **e**, Cell numbers (**d**) and percentages (**e**) of indicated cell types in scWAT of YT-FF and YT-iAKO mice fed HFD for 20 weeks. **f**, **g**, Cell numbers (**f**) and percentages (**g**) of indicated cell types in eWAT of mice as in (**d**). **h**, **i**, Cell numbers (**h**) and percentages (**i**) of indicated cell types in scWAT of aging mice. **j**, **k**, Cell numbers (**j**) and percentages (**k**) of indicated cell types in eWAT of aging mice. N=3 (**a** and **b**), 6 (**d**-**k**) mice per group, and data are mean ± s.e.m. Two-tailed unpaired Student's *t*-test (**d**-**k**). *$P < 0.05$, **$P < 0.01$, ***$P < 0.001$. Specific p-values and source data are provided as a Source Data file.

Supplementary Figure 11

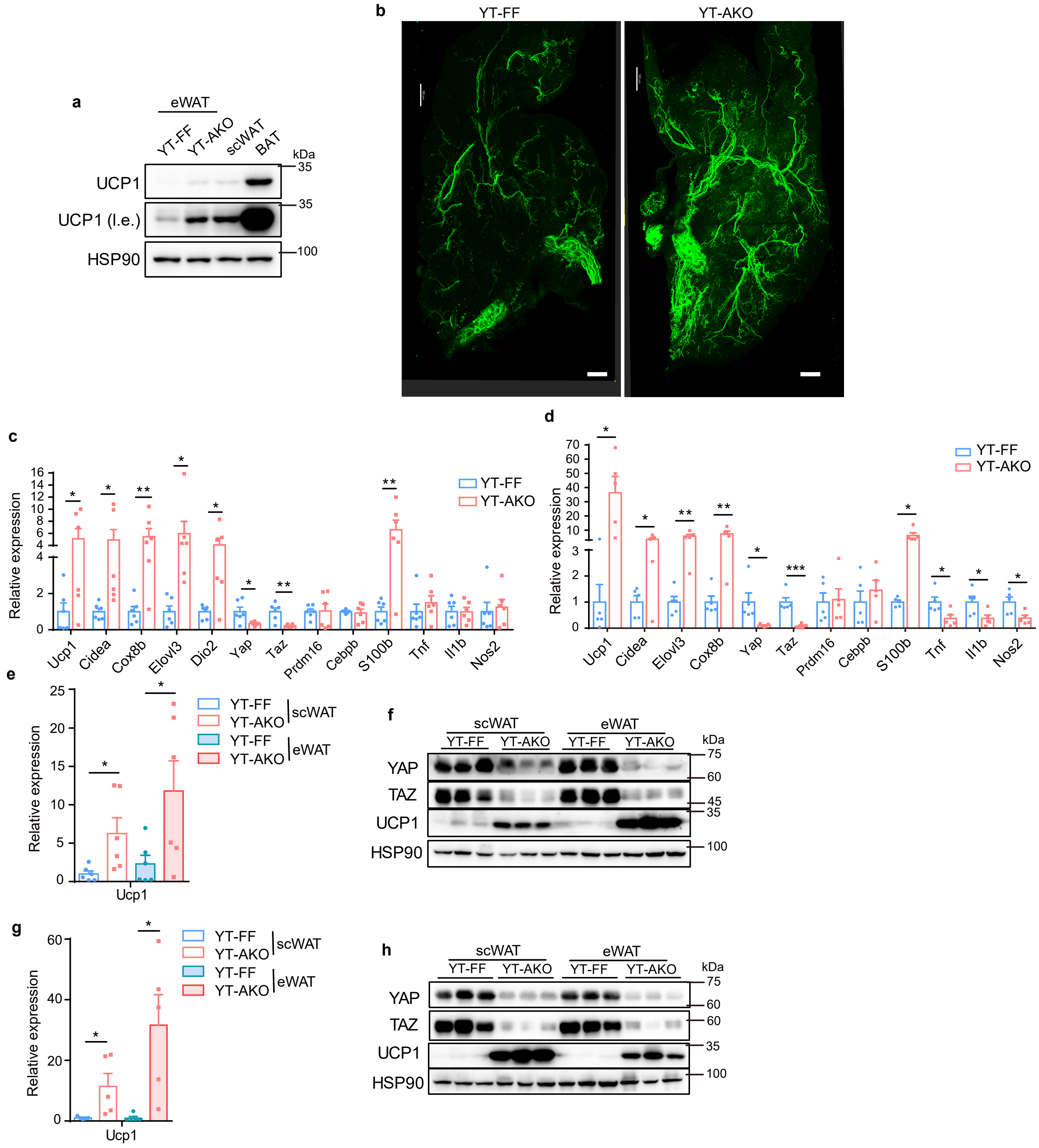

Supplementary Figure 11. Loss of *Yap/Taz* induces beige fat biogenesis in eWAT of age-associated and diet-induced obese mice. **a**, Immunoblot of UCP1 of eWAT from indicated mice under 4°C for 7 days, using scWAT and BAT lysate from mice under 22°C as positive controls. **b**, *Z*-stack images (400 μm) of TH staining of eWAT from adult YT-FF and YT-AKO mice. Scale bars: 0.5 mm. **c**, Relative mRNA levels of indicated genes in eWAT from 12-month-old YT-AKO and YT-FF male mice. **d**, Relative mRNA levels of indicated genes in eWAT from YT-AKO and YT-FF mice on HFD for 16 weeks. **e**, Relative mRNA level of *Ucp1* in scWAT and eWAT from mice in **c**. **f**, Immunoblot of indicated proteins in scWAT and eWAT from mice in **c**. **g**, Relative mRNA level of *Ucp1* in scWAT and eWAT from mice in **d**. **h**, Immunoblot of indicated proteins in scWAT and eWAT from mice in **d**. N=3 (**f** and **h**), 5 (**d** and **g**) or 6 (**c** and **e**) mice per group. Two-tailed unpaired Student's *t*-test (**c**-**e** and **g**). $*P < 0.05$, $**P < 0.01$, $***P < 0.001$. Specific p-values and source data are provided as a Source Data file.

Supplementary Figure 12

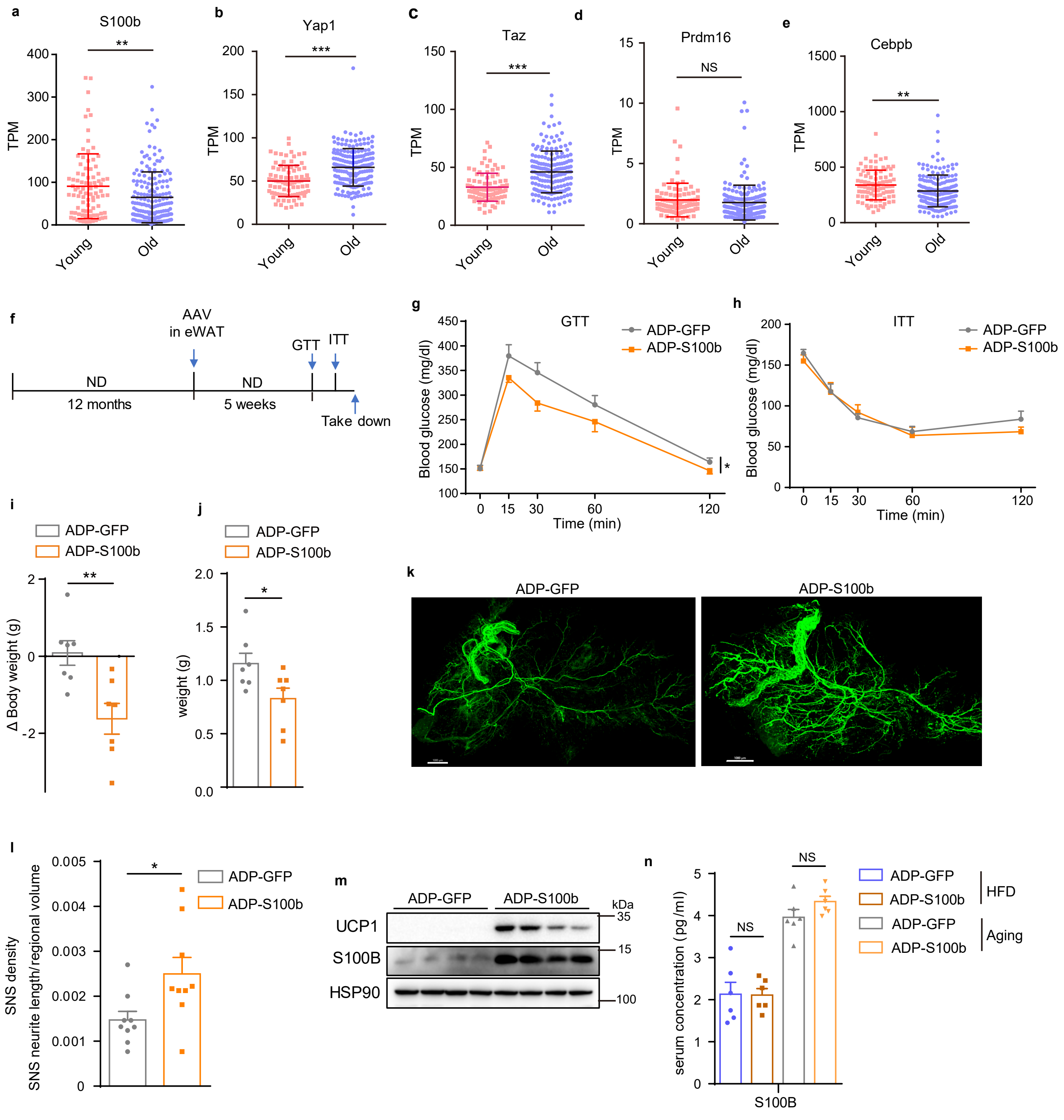

Supplementary Figure 12. AAV-*ADP-S100b* administration in eWAT relieves age-associated obesity and improves glucose homeostasis. **a**-**e**, mRNA abundance (TPM) of indicated genes in vWAT from young (20-39 years old, n=89) and old (60-79 years old, n=182) individuals, measured by RNA-seq from GTEx. **f**, Schematic diagram of AAV administration in aging mice. **g**, **h**, Glucose tolerance test (GTT) (**g**) and insulin tolerance test (ITT) (**h**) of mice as in (**f**). **i**, **j**, Weight gain of aging mice (**i**) and tissue weight of eWAT (**j**) upon AAV administration. **k**, **l**, Whole-mount TH immunostaining (**k**) and quantification (**l**) of cleared eWAT upon AAV administration. Scale bars: 1 mm. **m**, Immunoblot of indicated proteins in eWAT upon AAV administration. **n**, S100B level in sera. N=4 (**m**), 6 (**n**) or 7 (**g**-**j**) mice per group. Two-tailed unpaired Student's *t*-test (**a**-**e**, **i**, **j**, **l** and **n**); two-way ANOVA (**g** and **h**). *$P < 0.05$, **$P < 0.01$, ***$P < 0.001$; NS, not significant. Specific p-values and source data are provided as a Source Data file.

Supplementary Figure 13

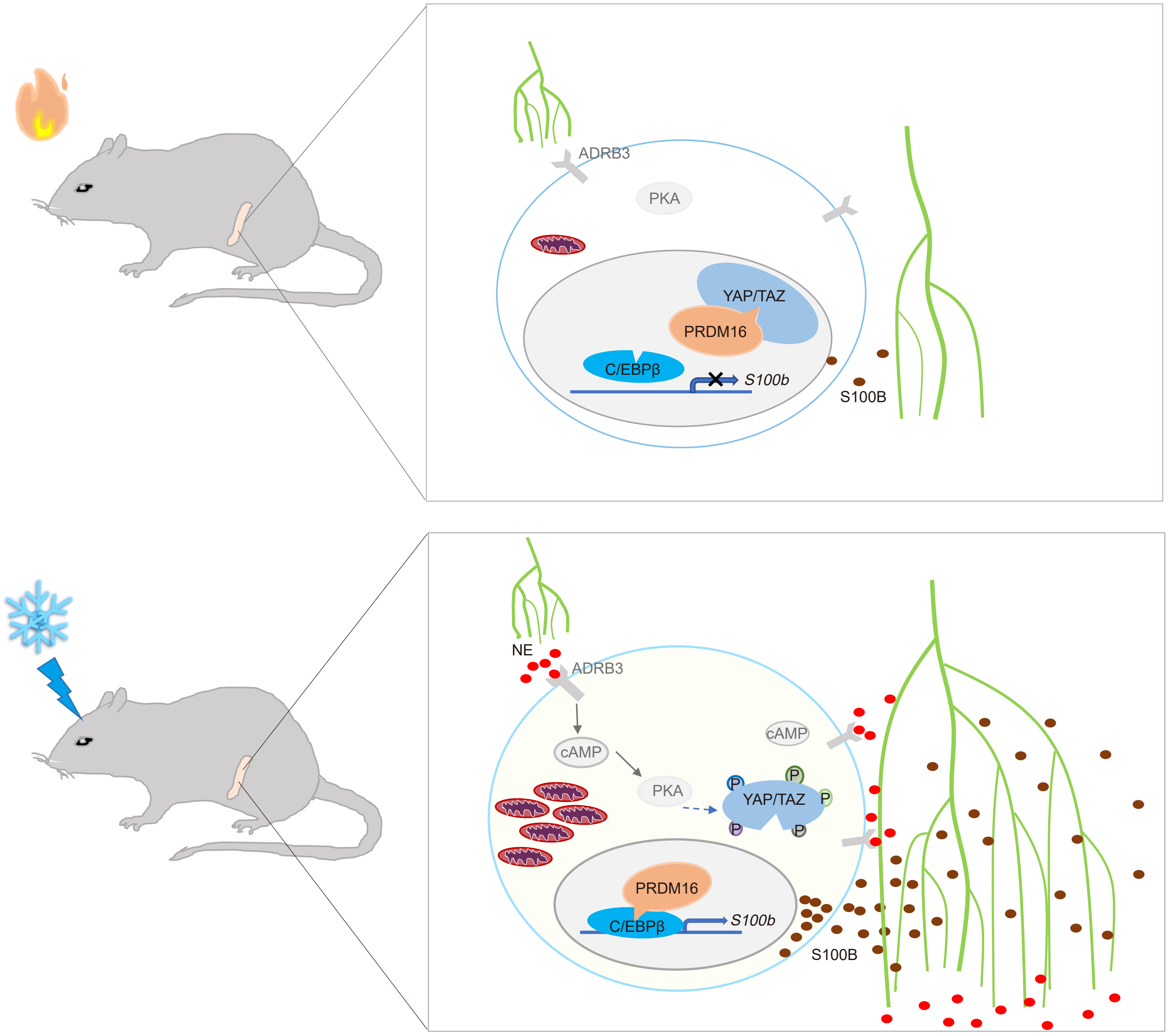

Supplementary Figure 13. The model showing how YAP/TAZ regulate sympathetic innervation in white adipose tissue. Under thermoneutrality, active YAP/TAZ enter the nuclei of adipocytes, interact with PRDM16 and thus impede PRDM16-C/EBPβ complex formation, which blocks the transcription of neurotrophic factor *S100b*. Upon cold exposure, sympathetic neurons secret noradrenaline (NE) to activate adipocyte ADRB3-cAMP-PKA signaling that phosphorylates and sequesters YAP/TAZ in the cytoplasm, releasing their inhibition on PRDM16-C/EBPβ complex formation. The PRDM16-C/EBPβ complex then binds to the promoter of *S100b* to drive its expression, which promotes sympathetic innervation and beige fat biogenesis.

Supplementary Table 1

gRNA:

**Gene**	**Forward (5'-3')**	**Reverse (5'-3')**
Yap-gRNA1	GATCAGACAACAACATGGC	GCCATGTTGTTGTCTGATC
Yap-gRNA2	ACCAGGTCGTGCACGTCCGC	GCGGACGTGCACGACCTGGT
Taz-gRNA1	TCACGTCATAGGACTGCTGG	CCAGCAGTCCTATGACGTGA
Taz-gRNA2	GCGCGAGTGCGAGCCGGAAT	ATTCCGGCTCGCACTCGCGC
S100b-gRNA	CCACCAGTACTCCGGGCGAG	CTCGCCCGGAGTACTGGTGG
Prdm16-gRNA	CCCGGGGCGTCACCACGTAG	CTACGTGGTGACGCCCCGGG
Cebpb-gRNA	TGGACGACGACGACGTGGAC	GTCCACGTCGTCGTCGTCCA

Supplementary Table 2

RT-qPCR-primers:

**Gene**	**Forward (5'-3')**	**Reverse (5'-3')**
Ucp1	ACTGCCACACCTCCAGTCATT	CTTTGCCTCACTCAGGATTGG
Cidea	TGCTCTTCTGTATCGCCCAGT	GCCGTGTTAAGGAATCTGCTG
Dio2	AATTATGCCTCGGAGAAGACCG	GGCAGTTGCCTAGTGAAAGGT
Cox8b	GAACCATGAAGCCAACGACT	GCGAAGTTCACAGTGGTTCC
Elovl3	GATGGTTCTGGGCACCATCTT	CGTTGTTGTGTGGCATCCTT
Prdm16	CAGCACGGTGAAGCCATTC	GCGTGCATCCGCTTGTG
Cebpb	ACGACTTCCTCTCCGACCTCT	CGAGGCTCACGTAACCGTAGT
Yap	TGAGATCCCTGATGATGTACCAC	TGTTGTTGTCTGATCGTTGTGAT

Taz	GCAGCAAGTCATCCACGTCA	AGGACTCCGGGAGGATCTTT
S100b	TGGTTGCCCTCATTGATGTCT	CCCATCCCCATCTTCGTCC
Adipoq	GCACTGGCAAGTTCTACTGCAA	GTAGGTGAAGAGAACGGCCTTGT
Ap2	ACACCGAGATTTCCTTCAAACTG	CCATCTAGGGTTATGATGCTCTTC
Pparg	CAAGAATACCAAAGTGCGATCAA	GAGCTGGGTCTTTTCAGAATAATAAG
Ngf	CCAGTGAAATTAGGCTCCCTG	CCTTGGCAAAACCTTTATTGGG
Bdnf	TCATACTTCGGTTGCATGAAGG	AGACCTCTCGAACCTGCCC
Ntf3	GGAGTTTGCCGGAAGACTCTC	GGGTGCTCTGGTAATTTTCCTTA
Ntf4	TGAGCTGGCAGTATGCGAC	CAGCGCGTCTCGAAGAAGT
Negr1	ACTTTGCGCCTACAATTCAGG	AACTTGCCAGGGAATGGAACT
Adrb1	GAACCCTGCAACCTGTCGTC	CCACGAGTAGGCCCATACC
Adrb2	GGGAACGACAGCGACTTCTT	GCCAGGACGATAACCGACAT
Adrb3	AGAAACGGCTCTCTGGCTTTG	TGGTTATGGTCTGTAGTCTCGG
Tnf	GTTCTATGGCCCAGACCCTCAC	GGCACCACTAGTTGGTTGTCTTTG
Il1b	AGCTTCCTTGTGCAAGTGTCT	GCAGCCCTTCATCTTTTGGG
Nos2	GAGCAACTACTGCTGGTGGT	TCAGAGTCTGCCCATTGCTG
