# Supplementary material for: Transcriptional repression of beige fat innervation via a YAP/TAZ-S100B axis
Source: Nat Commun. 2023 Nov 4;14:7102. doi: 10.1038/s41467-023-43021-8 (PMC10625615; doi:10.1038/s41467-023-43021-8)
Supplement: Supplementary file 3 — Description of Additional Supplementary Files [file 41467_2023_43021_MOESM3_ESM.pdf]

Description of Additional Supplementary Files

Supplementary Movie 1. **Circling phenotype of adult YT-UKO mice.** 8-week-old YT-UKO mice under room temperature exhibits circling behavior.

Supplementary Movie 2. **3D Visualization of sympathetic innervation in scWAT, related to Fig. 3a.** Part 1: TH immunolabeling in scWAT from 8-week-old YT-AKO mice. Part 2: TH immunolabeling in scWAT from 8-week-old YT-FF mice. The movie firstly shows a low-resolution 3D projection of the scWAT, followed by high-resolution 3D projection, 3D reconstruction and organization of the SNS parenchymal innervation.

Supplementary Movie 3. **Optical section of UCP1 immunostaining of eWAT, related to Fig. 7i.** Part 1: Sequential optical sections of UCP1 whole-mount imaging in an eWAT from 8-week-old YT-AKO mice after cold exposure for 1 week. Part 2: Sequential optical sections of UCP1 whole-mount imaging in an eWAT from 8-week-old YT-FF mice after cold exposure for 1 week.
