# Supplementary material for: Transcriptional repression of beige fat innervation via a YAP/TAZ-S100B axis
Source: Nat Commun. 2023 Nov 4;14:7102. doi: 10.1038/s41467-023-43021-8 (PMC10625615; doi:10.1038/s41467-023-43021-8)
Supplement: Supplementary file 7 — Reporting Summary [file 41467_2023_43021_MOESM7_ESM.pdf]

nature portfolio

Corresponding author(s): Yifu Qiu

Last updated by author(s): 2023-Oct-23

Reporting Summary

Nature Portfolio wishes to improve the reproducibility of the work that we publish. This form provides structure for consistency and transparency in reporting. For further information on Nature Portfolio policies, see our Editorial Policies and the Editorial Policy Checklist.

Statistics

For all statistical analyses, confirm that the following items are present in the figure legend, table legend, main text, or Methods section.

n/a	Confirmed	
☐	☒	The exact sample size (*n*) for each experimental group/condition, given as a discrete number and unit of measurement
☐	☒	A statement on whether measurements were taken from distinct samples or whether the same sample was measured repeatedly
☐	☒	The statistical test(s) used AND whether they are one- or two-sided<br>*Only common tests should be described solely by name; describe more complex techniques in the Methods section.*
☒	☐	A description of all covariates tested
☐	☒	A description of any assumptions or corrections, such as tests of normality and adjustment for multiple comparisons
☐	☒	A full description of the statistical parameters including central tendency (e.g. means) or other basic estimates (e.g. regression coefficient) AND variation (e.g. standard deviation) or associated estimates of uncertainty (e.g. confidence intervals)
☐	☒	For null hypothesis testing, the test statistic (e.g. *F*, *t*, *r*) with confidence intervals, effect sizes, degrees of freedom and *P* value noted<br>*Give P values as exact values whenever suitable.*
☒	☐	For Bayesian analysis, information on the choice of priors and Markov chain Monte Carlo settings
☒	☐	For hierarchical and complex designs, identification of the appropriate level for tests and full reporting of outcomes
☒	☐	Estimates of effect sizes (e.g. Cohen's *d*, Pearson's *r*), indicating how they were calculated

Our web collection on statistics for biologists contains articles on many of the points above.

Software and code

Policy information about availability of computer code

Data collection	Applied Biosystems StepOnePlus Real-Time PCR Systems, Tanon 5200 Chemiluminescent Imaging System, Comprehensive Lab Animal Monitoring System, Rectal temperature probe, Olympus BX51 Microscopy, BioTek Multi-Detection Microplate Reader, EVOS® FL Imaging Systems, GE Lunar PIXImus densitometer, Nikon AIR-si Microscopy, Zeiss Lightsheet Z.1, LiTone XL Light-sheet Microscope, Beckman Coulter CytoFLEX S flow cytometer, NovaSeq 6000 instrument, Orbitrap Q Exactive mass spectrometer.
Data analysis	ImageJ v10 was used for immunoblot densitometry analysis and cell area analysis, Imaris v9.9.1, GraphPad Prism v8.0.2, clusterProfiler R v3.18.1, HISAT2 v2.1.0, SAMtools v1.3.1, DEseq2 V2.11.40.6. CytExpert v2.3.0.84 , Flowjo v10

For manuscripts utilizing custom algorithms or software that are central to the research but not yet described in published literature, software must be made available to editors and reviewers. We strongly encourage code deposition in a community repository (e.g. GitHub). See the Nature Portfolio guidelines for submitting code & software for further information.

Data

Policy information about availability of data

All manuscripts must include a data availability statement. This statement should provide the following information, where applicable:

- Accession codes, unique identifiers, or web links for publicly available datasets
- A description of any restrictions on data availability
- For clinical datasets or third party data, please ensure that the statement adheres to our policy

The RNA seq data generated in this study has been deposited in the Gene Expression Omnibus (GEO) under accession number GSE222069 (https://

www.ncbi.nlm.nih.gov/geo/query/acc.cgi?acc=GSE222069) and are publicly available as of the date of publication. The ChIP-seq data used in this study were available in GEO under accession number GSE63965 (PRDM16) (https://www.ncbi.nlm.nih.gov/geo/query/acc.cgi?acc=GSE63965), GSM686970 (C/EBPβ) (https://www.ncbi.nlm.nih.gov/geo/query/acc.cgi?acc=GSM686970) and GSM1218859 (C/EBPα) (https://www.ncbi.nlm.nih.gov/geo/query/acc.cgi?acc=GSM1218859). The RNA seq data of WAT from human were available in GEO under accession number GSE141432 (https://www.ncbi.nlm.nih.gov/geo/query/acc.cgi?acc=GSE141432) and from GTEx database (https://www.gtexportal.org/home%5B33). The potential transcriptional factors binding to SP was predicted by PROMO (https://alggen.lsi.upc.es/cgi-bin/promo_v3/promo/promoinit.cgi?dirDB=TF_8.3/). Source data are provided with this paper.

Research involving human participants, their data, or biological material

Policy information about studies with human participants or human data. See also policy information about sex, gender (identity/presentation), and sexual orientation and race, ethnicity and racism.

Reporting on sex and gender	N/A
Reporting on race, ethnicity, or other socially relevant groupings	N/A
Population characteristics	N/A
Recruitment	N/A
Ethics oversight	N/A

Note that full information on the approval of the study protocol must also be provided in the manuscript.

Field-specific reporting

Please select the one below that is the best fit for your research. If you are not sure, read the appropriate sections before making your selection.

☒ Life sciences ☐ Behavioural & social sciences ☐ Ecological, evolutionary & environmental sciences

For a reference copy of the document with all sections, see nature.com/documents/nr-reporting-summary-flat.pdf

Life sciences study design

All studies must disclose on these points even when the disclosure is negative.

Sample size	The chosen of sample size was based on the pilot experiments or studies published (doi:10.1038/s41467-022-33800-0, doi:10.1038/s41590-021-01023-y) and to ensure statistical possibility, meanwhile to minimize animal usage in the experiments based on the 3R principles.
Data exclusions	No data was excluded.
Replication	All experiments were verified by biological replicates
Randomization	All experiments were randomly allocated to groups.
Blinding	Blinding was not done. A fully informed data analysis was performed.

Reporting for specific materials, systems and methods

We require information from authors about some types of materials, experimental systems and methods used in many studies. Here, indicate whether each material, system or method listed is relevant to your study. If you are not sure if a list item applies to your research, read the appropriate section before selecting a response.

Materials & experimental systems

n/a	Involved in the study	
☐	☒	Antibodies
☐	☒	Eukaryotic cell lines
☒	☐	Palaeontology and archaeology
☐	☒	Animals and other organisms
☒	☐	Clinical data
☒	☐	Dual use research of concern
☒	☐	Plants

Methods

n/a	Involved in the study	
☒	☐	ChIP-seq
☐	☒	Flow cytometry
☒	☐	MRI-based neuroimaging

Antibodies

Antibodies used	Antibodies for immunoblot<br>Primary Antibodies:<br>Rabbit anti-UCP1 Sigma-Aldrich #U6382. 1:5000<br>Rabbit anti-S100B Cell Signaling #9550. 1:1000<br>Rabbit anti-PKA substrate (RRXS*/T*) Cell Signaling #9624. 1:1000<br>Rabbit anti-PPARγ Cell Signaling #2435. 1:1000<br>Rabbit anti-C/EBPα Cell Signaling #2295. 1:1000<br>Mouse anti-HSP90α/β Santa Cruz Biotech #sc-13119. 1:10000<br>Mouse anti-C/EBPβ Santa Cruz Biotech #sc-7962x. 1:10000<br>Mouse anti-Lamin B1 Protein tech #66095-1-Ig. 1:10000<br>Mouse anti-α-tubulin Sigma-Aldrich #T6199. 1:10000<br>Rabbit anti-TH Millipore #AB1542. 1:3000<br>Sheep anti-PRDM16 R&D #AF6295. 1:400<br>Rabbit anti-YAP Cell Signaling #4912. 1:1000<br>Rabbit anti-TAZ Cell Signaling #4883. 1:1000<br>Rabbit p-YAP (Ser 112) Cell Signaling #4911. 1:1000<br>Rabbit p-TAZ (Ser 89) Cell Signaling #59971. 1:1000<br>Mouse anti-Flag Abmart #293881. 1:5000<br>Rabbit anti-HA Cell Signaling #3724. 1:1000<br>Rabbit anti-HSL Cell Signaling #4107. 1:1000<br>Rabbit anti-p-HSL (Ser 660) Cell Signaling #4126. 1:1000<br>Mouse anti-Perilipin1 Vala Science #4854. 1:10000<br>Mouse anti-p-Perilipin1 (Ser 522) Vala Science #4856. 1:10000<br><br>Secondary antibodies:<br>Goat anti-mouse IgG:HRP Thermo Scientific #32430. 1:10000<br>Goat anti-rabbit IgG:HRP Thermo Scientific #31460. 1:10000<br>Donkey anti-sheep IgG:HRP R&D #HAF016. 1:500<br><br>Antibodies for immunofluorescence<br>Rabbit anti-YAP Cell Signaling #14074. 1:100<br>Rabbit anti-TAZ Cell Signaling #83669. 1:100<br>Sheep anti-PRDM16 R&D #AF6295. 1:50<br>Rabbit anti-TH Millipore #AB1542. 1:100<br>Rabbit anti-UCP1 Abcam #ab10983. 1:100<br>Goat anti-Rabbit-IgG: Alexa Fluor 594 Abcam #ab150080. 1:300<br>Goat anti-sheep-IgG: Alexa Fluor 488 Invitrogen A11015. 1:400<br>Goat anti-Rabbit-IgG: Alexa Fluor 488 Cell Signaling #4412. 1:400<br><br>Antibodies for CUT&Tag<br>Sheep anti-PRDM16 R&D #AF6295. 1:50<br>Mouse anti-C/EBPβ Santa Cruz Biotech #sc-7962x. 1:100
Validation	All antibodies used were purchased from commercial vendors. Validation of these antibodies is described in Data Sheets which can be download from manufacturer's websites.

Eukaryotic cell lines

Policy information about cell lines and Sex and Gender in Research

Cell line source(s)	HEK 293T (CRL-3216) was obtained from American Type Culture Collection (ATCC).
Authentication	None of the cell lines have been authenticated.
Mycoplasma contamination	Frozen batch used for experiment was tested for mycoplasma and resulted negative.
Commonly misidentified lines (See ICLAC register)	No commonly misidentified cell lines were used in the study.

Animals and other research organisms

Policy information about studies involving animals; ARRIVE guidelines recommended for reporting animal research, and Sex and Gender in Research

Laboratory animals	Yap1fl/fl (027929, C57BL/6-129), AdipoqCre (028020, C57BL/6), Ucp1Cre (024670, C57BL/6), AdipoqCreERT2 (025124, C57BL/6), E2aCre (003724, C57BL/6) and Rosa26-LSL-Cas9 knock-in (026175, C57BL/6) mice were purchased from The Jackson Laboratories.

	Tazfl/fl mice (C57BL/6-129) were kindly provided by Nan Tang (National Institute of Biological Sciences, Beijing, China). Cas9Rosa26-LSL;AdipoqCre and Cas9Tg/Tg mice were generated by crossing Rosa26-LSL-Cas9 knock-in mice with AdipoqCre and E2aCre mice respectively. All mice were housed at temperature (22 ± 1 °C) and humidity (60% ± 10%) controlled environment under 12 h-12 h light-dark cycle. Unless specified, animals used in the experiment are 8-10 weeks old male mice.
Wild animals	This study did not involve wild animals.
Reporting on sex	Sex was considered in this study.The main conclusion of this study apply to both the male and female. We used male and female mice respectively to verify the YAP/TAZ-loss induced browning and S100B expression in scWAT. Besides, our SVF-derived beige adipocytes were mixture from male and female mice.
Field-collected samples	This study did not involve field-collected samples.
Ethics oversight	All procedures involving animals followed the protocols approved by the IACUC of Peking University and conformed to the Guide for the Care and Use of Laboratory Animals (IMM-QiuYF-1).

Note that full information on the approval of the study protocol must also be provided in the manuscript.

Flow Cytometry

Plots

Confirm that:

- [x] The axis labels state the marker and fluorochrome used (e.g. CD4-FITC).
- [x] The axis scales are clearly visible. Include numbers along axes only for bottom left plot of group (a 'group' is an analysis of identical markers).
- [x] All plots are contour plots with outliers or pseudocolor plots.
- [x] A numerical value for number of cells or percentage (with statistics) is provided.

Methodology

Sample preparation	Inguinal scWAT or eWAT was minced and digested with Collagenase Type I (Worthington, 180 U/ml) in SVF buffer (118 mM NaCl, 1.1 mM CaCl2, 2.7 mM KCl, 0.5 mM MgCl2, 0.4 mM NaH2P04, 20 mM HEPES, 5.5 mM Glucose, 1% BSA) at 37°C with agitation at 150 rpm for 30 min. The digested cell suspension was passed through a 40-μm strainer and pelleted cells were resuspended in red blood cell lysis buffer (0.15 M NH4Cl, 1.1 mM EDTA, 10 mM NaHC03,) for 5 min at RT to remove blood red cells. Finally, the cell pellet was resuspended in FACS buffer (PBS containing 2% FBS and 1 mM EDTA) for further staining.
Instrument	Beckman Coulter CytoFLEX S
Software	CytExpert v2.3.0.84 , Flowjo v10
Cell population abundance	The abundance of cell populations was presented in the graphs in Extended Data Fig. 10d-k
Gating strategy	We first gated the singlets by FSC-A and FSC-H, and exclude dead cells by gating PB450 negative cells . Specific cell populations were determined by markers listed below Macrophages: CD45+/F4/80+; M1 macrophages: CD45+/F4/80+/CD11c+/CD206-; M2 macrophages: CD45+/F4/80+/CD11c-/ CD206+

- [x] Tick this box to confirm that a figure exemplifying the gating strategy is provided in the Supplementary Information.
